# A Systematic Review of the Health and Social Effects of Menstrual Hygiene Management

**DOI:** 10.1371/journal.pone.0062004

**Published:** 2013-04-26

**Authors:** Colin Sumpter, Belen Torondel

**Affiliations:** Department of Disease Control, London School of Hygiene and Tropical Medicine, London, United Kingdom; Tehran University of Medical Sciences, Iran (Islamic Republic Of)

## Abstract

**Background:**

Differing approaches to menstrual hygiene management (MHM) have been associated with a wide range of health and psycho-social outcomes in lower income settings. This paper systematically collates, summarizes and critically appraises the available evidence.

**Methods:**

Following the PRISMA guidelines a structured search strategy was used to identify articles investigating the effects of MHM on health and psycho-social outcomes. The search was conducted in May 2012 and had no date limit. Data was extracted and quality of methodology was independently assessed by two researchers. Where no measure of effect was provided, but sufficient data were available to calculate one, this was undertaken. Meta-analysis was conducted where sufficient data were available.

**Results:**

14 articles were identified which looked at health outcomes, primarily reproductive tract infections (RTI). 11 articles were identified investigating associations between MHM, social restrictions and school attendance. MHM was found to be associated with RTI in 7 papers. Methodologies however varied greatly and overall quality was low. Meta-analysis of a subset of studies found no association between confirmed bacterial vaginosis and MHM (OR: 1.07, 95% CI: 0.52–2.24). No other substantial associations with health outcomes were found. Although there was good evidence that educational interventions can improve MHM practices and reduce social restrictions there was no quantitative evidence that improvements in management methods reduce school absenteeism.

**Conclusion:**

The management of menstruation presents significant challenges for women in lower income settings; the effect of poor MHM however remains unclear. It is plausible that MHM can affect the reproductive tract but the specific infections, the strength of effect, and the route of transmission, remain unclear. There is a gap in the evidence for high quality randomised intervention studies which combine hardware and software interventions, in particular for better understanding the nuanced effect improving MHM may have on girls’ attendance at school.

## Introduction

Menstruation is a natural and beneficial monthly occurrence in healthy adolescent girls and pre-menopausal adult women. It concerns women and men alike as it is among the key determinants of human reproduction and parenthood. The age of menarche varies by geographical region, race, ethnicity and other characteristics but ‘normally’ occurs in low income settings between the ages of 8 and 16 with a median of around 13. [1], [2] The median age of menopause is estimated at around 50 years. [3] By using these figures we can calculate that between menarche and menopause a woman in a low income country may expect to menstruate for around 1400 days in her lifetime.

Globally women and girls have developed their own personal strategies to cope with menstruation. These vary greatly from country to country, and within countries, dependent on an individual's personal preferences, available resources, economic status, local traditions and cultural beliefs and knowledge or education. Due to these restrictions women often manage menstruation with methods that could be unhygienic or inconvenient, particularly in poorer settings.

Estimates of the prevalence of methods of management vary greatly across contexts but studies report widespread use of unsanitary absorbents, and inadequate washing and drying of reused absorbents across Africa, South East Asia and the Middle East. Studies in Africa have found use of sanitary pads as low as 18% amongst Tanzanian women with the remainder using cloth or toilet paper. [4] Studies of Nigerian schoolgirls have found between 31% and 56% using toilet tissue or cloth to absorb their menstrual blood as oppose to menstrual pads. [5], [6] A study of women in Gambia found that only around a third regularly used sanitary pads. [7] Studies in India have found between 43% and 88% of girls washing and reusing cotton cloth rather than using disposable pads. [8], [9] It has been found that cleaning of cloths is often done without soap or with unclean water and drying may be done indoors rather than in sunlight or open air due to social restrictions and taboos. These practices may lead to reuse of material that has not been adequately sanitised. [9] Across studies problems are found to be particularly acute in rural areas and amongst women and girls in lower socio-economic groups.

The burden of reproductive tract infections (RTI) is a major public health concern worldwide and RTI are particularly widespread in low income settings. [10], [11] The proportion of this burden that can be attributed to poor menstrual hygiene management (MHM), as opposed to sexually transmitted infections; iatrogenic infections; or endogenous infections caused by agents other than those introduced through poor menstrual management is unknown. Confusing any attempt to investigate this is the fact that concurrent infection from multiple sources is possible. RTIs thought to be of most relevance to MHM are the endogenous infections bacterial vaginosis (BV) and vulvovaginal candidiasis (VVC). These vaginal imbalances are primarily non-sexually transmitted and could plausibly be introduced to the reproductive tract through the materials used for absorbing menstrual blood or by poor personal hygiene during the menstrual period. BV has been associated with an increase risk of HIV infection [12], [13]; human papillomavirus infection [14] and with adverse pregnancy outcomes [15] amongst others. Vulvovaginal candidiasis has also been associated with HIV infection. [16] BV and VVC have similar symptomatic displays with vaginal discharge and irritation although many infections remain asymptomatic.

Across the globe menstruation and its management also have important social and cultural implications which may in turn impact women and girls' lives. In some cultures girls become marriageable and regarded as moving their role to child bearing with the onset of menstruation. [17], [18] The sexual and disgust connotations of menstruation make it a taboo subject for girls to raise, even with their mothers. Without good information, young girls may be frightened at the onset of their period and may be anxious about the process. [6] A qualitative study found that two thirds of South Indian girls described their menarche as shocking or fearful. In the study setting menarche was also ‘celebrated’ with a 9 to 13 day seclusion period with many behavioural restrictions. [9] Following menarche the social effects of the ineffective management of regular menstruation may include exclusion from everyday tasks including touching water, cooking, cleaning, attending religious ceremonies, socialising, or sleeping in one's own home or bed. [17]–[23]

Absence from school, or drop-out from school, has been of particular interest to International organisations and research bodies working in this area such as WaterAid, the Water Research Commission and Plan International. These organisations report that in their experience girls' absence from school during menstruation can have both physical and psychological causes. [20], [24] First, they may lack physical provisions for MHM such as lockable, single-sex, private toilets with water and soap for washing, a private open air space to dry wet cloths and a closed bin or incinerator for used pads. Menstrual pain is another reason for girls to go absent themselves. [20], [24] Girls have also reported feelings of fear, confusion and shame in class due to: leakage and dropping of sanitary material; smell and staining of clothes; teasing, fears of pregnancy; and experience of harassment by male students and teachers [20],[23]–[25]

It has been reported that females staying longer in school is associated with reduced maternal death; improved population health; increased contraceptive uptake; decreased fertility rate, improved child health; increased vaccination rates and decreased infection rates with HIV. [26] Interventions that increase years of schooling may clearly have important secondary health outcomes and wider economic benefit.

### Objective of the review

The objective of this review is to collate, summarize and critically appraise the peer-reviewed and published evidence on the health and psycho-social outcomes of the methods of MHM used in low and middle income countries and to assess the evidence for existing interventions such as educational programs and absorbent distribution.

No protocol of the review is available on-line but all methods are outlined in this paper and further detail is available from the corresponding author.

## Materials and Methods

### Search Strategy

**Figure 1:**
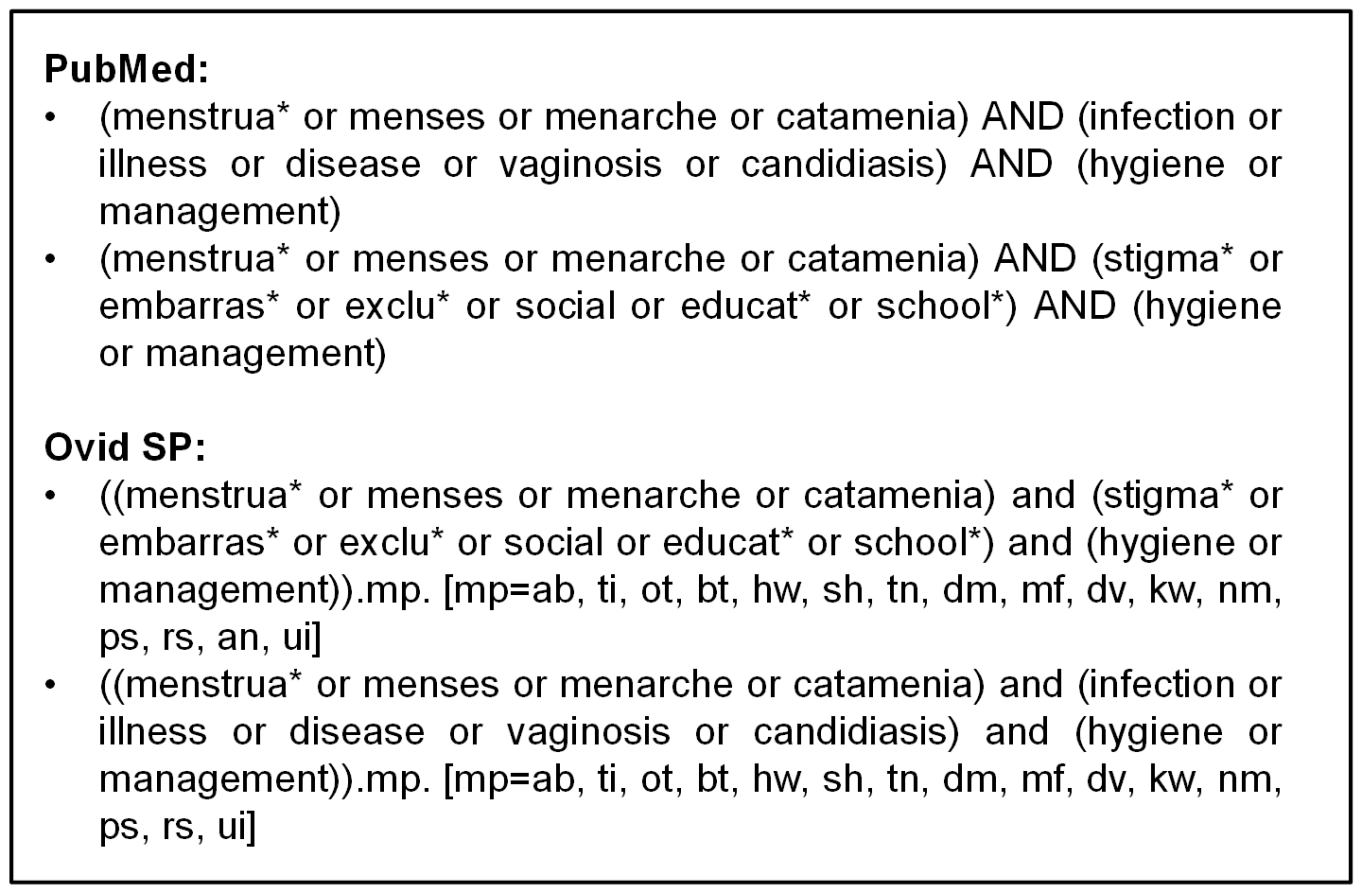
Search terms.

Our search strategy was designed to identify published studies on MHM and associated health or social outcomes. We were interested in locating studies which looked at any method of MHM or behaviour. We sought to identify both intervention studies and observational evidence. The searches were conducted in May 2012 using three on-line databases: Medline through PubMed; CAB Abstracts; Embase and Global Health through Ovid SP. No date limit was set on the search to ensure as wide a range of articles were identified as possible. In addition each paper included in the review was hand-searched for additional references. Search terms were generated to encapsulate the four main concepts our review pertains to: menstruation; and hygiene/management; health effects; and social effects. Figure 1 shows these terms and how they were combined.

### Inclusion Criteria

#### Studies

To be eligible papers were required to be: published in a peer reviewed journal; written or translated into English; available in the public domain; and be original primary research including experimental, observational and qualitative studies but excluding economic analyses, systematic reviews, project reports, policy analysis and other commentary. Purely descriptive studies, for example those focusing only on proportion of women using various management methods but not associating these with health or social outcomes, were excluded. Studies with a primary focus on management in high-income countries including tampon use (and associated infections and toxic shock syndrome) and talcum powder (and its associations with ovarian cancer) were excluded i.e. those that did not feature unhygienic menstrual protection as an exposure variable in their analysis but rather compared two alternative, but relatively hygienic, methods. Although these areas may be worthy of further investigation in the developing country context they were felt to be outside the scope of this review.

#### Participants

All papers were required to include analysis relevant to menstruating females from low and middle income countries. No other restriction was set on study participants beyond this requirement. Low and middle income settings were chosen as they are the settings where the lack of available resource to maintain menstrual hygiene is highest.

#### Exposures

Papers were required to include a clear description of the menstrual management methods under investigation. Based on prior reading unhygienic or poor menstrual management methods were likely to include inadequate washing or drying of reusable pads and the use of disposable cloth rags or other absorbents. Interventions aimed at reducing social restrictions or poor menstrual practices included educational interventions and pre-menarcheal training.

#### Outcomes

All papers were required to investigate the extent to which menstruation or menstrual management were associated with health or social outcomes. The health outcomes of interest specified in the search terms were left purposefully wide in order to capture all the potential infections and diseases, which included reproductive tract infections (including bacterial vaginosis and vulvo-vaginal candidiasis), other reproductive infections (secondary infertility), urinary tract infections and anaemia. The social outcomes of interest were social restrictions such as limiting diet or interactions during menstruation and school absenteeism.

#### Data extraction and quality assessment

For this review an initial screen of titles and abstracts was done online to ensure that included papers broadly reflected the initial inclusion and exclusion criteria. Foreign language papers identified through English abstracts were assessed before exclusion to minimise the potential for any foreign language bias. When a title and abstract could not be rejected with certainty, the abstract was downloaded for more detailed scrutiny using the initial inclusion and exclusion criteria. Where papers could not be clearly rejected using the abstracts, the full text of the article was obtained for full scrutiny. Once the list of abstracts was ready, as many papers in full text as possible were obtained. Papers were examined to ensure that they did not display the same data set in different papers.

Following the complete search, data were extracted from the identified studies using pre-designed tables to allow cross-study comparison. Studies were critiqued for rigour using checklists adapted from the Critical Appraisal Skills Programme (CASP) and tabulated to allow comparison of quality issues across the body of evidence.

Data extracted from the papers included study type, population and sample size, the menstrual practices investigated, the outcome measure used and the measure of effect reported including any adjustments made. Where no measure of effect was provided available raw data was taken from the paper and a crude odds ratio was calculated using Stata. Where this was done it is clearly marked in results tables.

The high heterogeneity in approach, in particular the differing measures of exposure and differences in populations studied, mean there was little value in calculating a pooled OR across all studies. We attempted to reduce this heterogeneity by looking at a subset of studies with comparable methods. The odds ratios of exposure to poor menstrual practices among those women with confirmed bacterial vaginosis compared to women without confirmed bacterial vaginosis were plotted using a forest plot and a pooled odds ratios (OR) was calculated. The DerSimonian and Laird random effects method was used to allow for our understanding that any effect of menstrual hygiene on bacterial vaginosis is likely to vary between studies due to study contexts, participants and type of intervention used. [27]

Our review was conducted in line with the requirements of the Preferred Reporting Items for Systematic Reviews and Meta-Analyses statement (PRISMA) and a checklist was completed at conclusion of the review. [28]

## Results

### Available Evidence

The search returned 4135 articles through Medline; CAB Abstracts; Embase; and Global Health. These were catalogued using EndNote referencing software. An initial screen identified 2211 duplicate entries due to searches being repeated across multiple databases. These duplicates were excluded.

Through title and abstract review 1859 articles were removed because they did not offer any analysis regarding associations with MHM. These included studies focused on contraception use (250); cancer (176); hormonal replacement therapy/menopause (173); migraine (44); nutritional status (41); age at menarche (34); abortion (22); neonatal outcomes (24); and the impact of athletic pursuits (18); amongst others. In addition medical guidelines and articles on clinical management resulted in a further 255 papers being discarded.

The remaining 65 articles were reviewed in full and a further 46 were rejected. Many studies (29) were identified that contained some discussion of menstruation or menstrual practices but contained no relevant analysis associating hygiene or menstrual management with social or health outcomes. These were primarily knowledge, attitudes and practice (KAP) studies and were used to inform the background of the work. Other rejected papers did not report any analysis relevant to the low income context (13); or had full text in a foreign language (Cantonese: 2; Turkish: 1).

**Figure 2:**
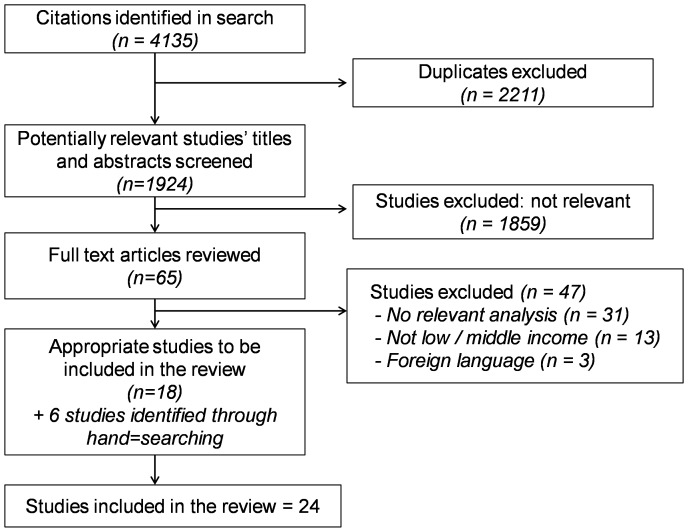
PRISMA flowchart.

Following the hand search of the bibliographies of reviewed papers 6 further articles were identified and included. In total 25 articles were included in the systematic review: 14 of these related to health effects; and 11 related to social effects including exclusion from activities and attendance at school. Figure 2 illustrates the process.

### Effects on Health

#### Study design, setting and population

Of the 14 identified articles 11 health outcome studies were cross-sectional, two were case-control and one was a cross-over intervention. The intervention study employed a cross-over design where each woman was followed for four menstrual cycles and received sanitary pads during 2 cycles and employed traditional methods for the other 2 cycles. [29]

Studies were conducted in a diverse range of settings. Half were conducted in urban areas (7/14) and half in rural (7/14). The majority of studies recruited subjects from health care settings (8/14), with the remainder going to the community (5/14), and in one study from a school (1/14). The majority of studies were conducted on the Indian subcontinent (7/14) but also include: sub-Saharan Africa (3/14); North Africa/Middle East (2/14); and China (1/14). The majority of studies were conducted amongst adult women aged around 15 to 50 years (12/14) with one conducted amongst school age girls only. Sample sizes ranged from 227 up to 3600 for cross-sectional studies.

Menstrual management was rarely the primary focus of the research however and this resulted in a number of included studies which are only relevant to specific populations such as HSV-2 positive women [4]; those currently using birth control [10] or ever having used birth control [30]; and those currently experiencing symptoms of RTI. [7]

#### Outcomes and exposures

All (14/14) identified articles looking at health outcomes used self-reported menstrual management as the exposure. Papers primarily compared type of absorbent used e.g. rags vs. disposable pads (9/14) but a minority compared the methods of washing of cloths used for absorption (3/14) or a composite hygiene index (2/14). A very wide range of definitions of ‘good’ and ‘bad’ MHM were used and no consistent standard was apparent. The use of disposable sanitary pads was considered as a good hygienic practice in all the papers which reported their use as an absorbent. Reusable cloths were considered bad practice when compared with disposable pads in some studies. [4], [7], [9], [29]–[32] But they were considered as a good MHM when they were washed hygienically and dried in the sun [33], [34] and compared with those not washed or dried inside. Some papers reported not using any absorbent as a negative practice [9], [31] but one study reported that not using any absorbent but staying at home and cleaning with the corner of the sari was more hygienic than using rags washed in apparently unclean river water. [10] The use of cotton, cotton wool or toilet paper as a menstrual absorbent was considered as a bad hygienic practice compared to disposable pads. [4], [34]

In terms of health outcome studied 11/14 studies had reproductive tract infection (RTI) endpoints. Over half of these (6/11) employed clinically or laboratory confirmed bacterial vaginosis (BV) and the remainder (5/11) relied on self-reported vaginal discharge. Case-control studies solely addressed secondary infertility and one cross-sectional looked at multiple outcomes including UTI and anaemia. No studies looked at confirmed vulvovaginal candidiasis or other specified RTIs.

#### Study quality and risk of bias

Standardised quality assessment summaries are provided in: table 1 for intervention studies; table 2 for case-control studies; and table 3 for cross-sectional studies.

**Table 1:** Quality assessment – Interventions (Health and Social).

	Groups				Blinding			Follow-up				Analysis				
First Author (Year)	Arms	Sample	Random^1^	Balance^2^	Subjects	Investigator	Analysis	Objective^3^	LTFU^4^	ITT^5^	Identical^6^	Power^7^	Effect size^8^	CI^9^	P-Value^10^	
***Health***																
Morison (2004)	1^a^	30	N	n/a	N	N	N	Y	Y	Y	Y	N	Y	Y	Y	
***Social***																
Fetohy (2007)	2^b^	124∶124	N^c^	N^d^	N	N	N	N^e^	Y^f^	Y	Y	N	N^g^	N	Y	
Fakhri (2012)	2^b^	349∶349	N^h^	N^i^	N	N	N	N^e^	Y^f^	Y	Y	N	N^j^	N	Y	
Nemade (2009)	1^k^	217	N	Y^l^	N	N	N	N^e^	Y^f^	n/a	Y	N	N^j^	N	Y	
Allah (2011)	1^k^	150	Y	Y^l^	N	N	N	N^e^	Y^f^	n/a	Y	Y	N^j^	N	Y	
Posner (2009)	1^k^	504	N	Y^m^	N	N	N	N^e^	Y^n^	n/a	Y	N	Y^o^	N	Y	
Dongre (2007)	1^k^	383	Y	Y	N	N	N	N^e^	n/a^p^	n/a	Y	N	N	Y	Y	
Oster (2010)	2	99∶99	Y	N	N	N	N	Y^q^	Y	Y^r^	Y	Y	Y	Y	Y	

1 Allocation/sampling process described and truly random; ^2^Comparison group characteristics provided and balanced; ^3^Objective measures of outcome used;^ 4^Number of subjects lost to follow up (LTFU) provided and analysed; ^5^Intention to Treat (ITT) analysis used; ^6^Identical follow-up in each arm; ^7^Power calculation provided; ^8^Measure of effect provided (e.g. OR/RR); ^9^Confidence Intervals provided; ^10^P-Value provided.

a Cross-over intervention; ^b^Two arms but employed post-test only; ^c^Cluster randomised, 10 classes (5 intervention, 5 control) but no randomisation process described; ^d^Intervention group significantly older; ^e^Self-reported approaches to menstrual management; ^f^Although not stated, numbers in analysis shows no loss to follow up; ^g^Difference in test score means, t-test and SD provided; ^h^Schools chosen to take part, controls matched on school type, grade, age, field of study; ^i^Other than matching no data on ‘balance’ presented; ^j^Difference in proportions (Chi^2^/t-test) and p-value provided; ^k^Before and after study; ^l^Same students interviewed pre- and post-intervention; ^m^Identical panel used: interviewed pre- and post-intervention; ^n^LTFU figure given, analysis of characteristics provided; ^o^Regressions model shows effect size, SE provided; ^p^Two independent samples used – no loss to follow up possible; ^q^Both self-reported and official records used to measure attendance; ^r^adherence c. 60%, ‘treatment on treated’ analysis presented separately.

**Table 2:** Quality assessment – Case-Control (Health only).

	Cases					Controls			Exposure (MHM)				Analysis			
First Author (Year)	Definition^1^	Sample	Represent^2^	Power^3^	System^4^	Represent^2^	Non-resp^5^	System^4^	Accurate^6^	Identical^7^	Blinding^8^	Temporal^9^	Confound^10^	Effect Size^11^	CI^12^	P-Value^13^
***Health***																
Ali (2007)	Y	400∶400	N^a^	Y	Y	Y	N^b^	Y	N^e^	Y	N	Y	N^c^	Y	Y	Y
Sami (2012)	Y	400∶400	N^a^	Y	Y	Y	Y^d^	Y	N^e^	Y	N	Y	N^f^	Y	Y	Y

1 Valid case definition provided?; ^2^Cases/controls representative of the general population e.g. community recruitment?; ^3^Power calculation; ^4^Systematic selection process used to select cases/controls e.g. matching, random selection, population based; ^5^Refusal to participate reported and reasonable; ^6^Exposure accurately measured; ^7^Exposure measured identically in cases and controls; ^8^Blinding used; ^9^Direction of the relationship explained; ^10^Appropriate confounders measured and accounted for; ^11^Measure of effect provided (e.g. OR/RR);^12^Confidence Intervals provided; ^13^P-Value provided.

a Recruitment at infertility clinics; ^b^Unclear how many controls refused participation; ^c^Amongst many other potential confounders, sexual behaviour, a common cause of RTIs, not accounted for; ^d^Low (3%) refusal rate; ^e^Self-reported menstrual hygiene; ^f^Although data collected on STIs, not clear if OR adjusted.

**Table 3:** Quality assessment – Cross-sectional studies (Health and Social).

	Selection					Outcomes		Co-factors (inc. MHM)			Analysis			
First Author (Year)	Sampling^1^	Study Size	Refusals^2^	Power^3^	Represent^4^	Definition^5^	Measured^6^	Range^7^	Definition^5^	Measured^8^	Adjustment	Effect size^9^	CI^10^	P-Value^11^
***Health***														
Bulut (1997)	N^a^	867	Y^b^	N	N^a^	Y	N^c^	Y	Y	N	N^d^	N	N	N
Wasserheit (1989)	Y^e^	2929	Y	N	N^f^	Y	Y^g^	Y	Y	N	Y^h^	Y	Y	N
Younis (1993)	Y	370	Y	Y	Y	Y	Y^g^	Y	Y	N	Y	Y	N	Y
Bhatia (1995)	N	3600	Y	N	Y	Y	N^i^	Y	Y	N	Y	Y	N	Y
Narayan (2001)	N	619	Y	N	Y	Y	N^j^	Y	Y	N	Y	Y	Y	N
Xia (2004)	Y	606	Y	Y	Y	Y	N	Y	Y	N	Y	Y	Y	Y
Baisley (2009)	Y	1305	Y	Y	N^k^	Y	Y^g^	Y	Y	N	Y	Y	Y	Y
Bahram (2009)	Y	500	N	Y	N	Y	Y^g^	Y	Y	N	N	N	N	Y
Demba (2005)	Y	227	N^l^	N	N	Y	Y	Y	Y	N	N	N	N	Y
Singh (2011)	Y	965	N	N	Y	Y	N	Y	Y	N	N	N	N	Y
Balamurugan (2012)	Y	656	N	Y	Y	Y	Y	Y	Y	N	N	N	N	Y
***Social***														
Aniebue. (2009)	Y	495	Y	Y	Y	Y	N^m^	Y	Y	N^n^	N	N	N	Y
Ali (2010)	Y	1275	N	Y	Y	Y	N^m^	Y	Y	N	N	N	N	Y

1 Simple random sampling (SRS) or reasonable alternative where SRS not possible; ^2^Response rate provided and explained;^ 3^Power calculation provided; ^4^Sample representative of wider population of interest to MHM (selection bias); ^5^Outcome/co-factor clearly defined; ^6^Outcome collected appropriately (misclassification bias); ^7^Suitable range of variables collected; ^8^Clear methods explained for collection (misclassification bias); ^9^Measure of effect provided (e.g. OR/RR); ^10^Confidence Intervals provided; ^11^p-values provided.

a Systematic recruitment from 11 year old register from clinic, sexually inactive women excluded; ^b^72% response rate but comparison provided; ^c^RTIs and UTIs clinically confirmed but self-reported outcomes used despite confirmed over-reporting; ^d^No adjustments made for sexual activity or any other factors collected; ^e^Cluster random sample: randomised health workers, not individuals; ^f^Sample designed to understand population using contraception; ^g^Clinical/laboratory confirmation of RTIs; ^h^Adjusted by contraceptive method, no other factor found to confound relationship; ^i^Self-reported or ‘perceived’ health problems; ^j^Self-reported ‘white discharge’; ^k^Herpes Simplex Positive ‘facility’ workers only; ^l^Consecutive recruitment from all genitor-urinary clinic attendees but no data on refusals; ^m^Self –reported menstrual management; ^n^Self-reported pre-menarcheal training.

Some common methodological limitations were identified in the body of health evidence. The majority of evidence (10/11 studies for RTI) lies in observational cross-sectional data so we cannot determine causality of the observed observations. These studies are open to confounding and can present issues such as reverse causality i.e. that an individual may have changed their menstrual practice due to an infection or other ailment rather than the management method caused the infection or ailment.

Studies primarily relied on subjective exposure or outcome measures such as self-reported hygiene (14/14), and many relied on self-reported health outcomes (6/14). Papers that reported on BV confirmed the cases using Nugent scores or Amsel criteria and a minority of studies which reported general RTIs diagnosed the infection clinically. [4], [7], [10], [29], [32], [35], [36] Self-reported information about menstruation management and health outcomes is likely to be subject to reporting bias as in most countries menstruation is a taboo and participants may prefer not to answer questions on this topic. Evidence from a study which followed up self-reporting with clinical confirmation demonstrated that self-reported symptoms are likely to be overestimates. [36]

The body of evidence also suffers greatly from the lack of standardisation with varying methods being used to categorise menstrual management, making comparison between studies extremely difficult.

Finally, there is limited adjustment for confounding with many studies (6/14) failing to adjust for any factor. This is likely to be due to the fact that MHM is considered only as a confounding factor at the outset of these studies, rarely as a primary investigative issue.

### Measures of Effect

Summaries of the results of the studies looking at health outcomes are provided in table 4. This table is divided into three subsections for positive associations, negative associations and null findings. Where a study reported more than one relevant finding these are reported on separate rows.

**Table 4:** Associations between menstrual practices and health outcomes.

First Author (year)	Setting	Design	Population and sample size		‘Good’ MHM Practice (Baseline)	‘Poor’ MHM Practice(Comparator)		Outcome		Measure of Effect^1^			Adjustments made	
***Positive Association***														
Narayan (2001)	IndiaSchoolUrban + Rural	Cross-sectional	12–17 yrsSchoolgirlsN = 619		‘High’ hygiene index score^2^	‘Low’ hygiene index score^2^		Self-reported white discharge		OR: 2.1(1.2–6.3)p<0.05			Socio-economic, rural-urban residence and age	
Baisley (2009)	TanzaniaHealth centreRural	Cross-sectional	16–35 yrsHSV-2 positive ‘facility’ workersN = 1305		Usually use sanitary pads	Usually use cloths/underwear/sponges		Clinically confirmed BV (Nugent score)		OR: 1.34(0.97–1.85) p = 0.02			Age, facility, dependents, alcohol, age at first sex, hormonal contraception, sex in last week	
Baisley (2009)	TanzaniaHealth centreRural	Cross-sectional	16–35 yrsHSV-2+‘facility’ workersN = 1305		Usually use sanitary pads	Usually use cotton wool/toilet paper		Clinically confirmed BV (Nugent score)		OR: 2.52(1.23–5.24)p = 0.02			Age, facility, dependents, alcohol, age at first sex, hormonal contraception, sex in last week	
Younis (1993)	EgyptHealth centreRural	Cross-sectional	Reproductive ageEver marriedN = 370		Boiling cloth or napkin used or using disposable	Washing cloth or pad with water and soap alone		Clinically confirmed RTI (non-specific)		OR: 1.66(No CI)^4^p<0.05			Age, education and socio-economic status	
Balamarugan (2011)	IndiaCommunity Urban	Cross-sectional	15–45 yrsAll womenN = 265		Only use sanitary pads	Use sanitary pads and cloths/cloths only		Clinically diagnosed BV(Amsel criteria)		OR:3.41 ^3^(1.2–10.1)P = 0.0182			None	
Singh (2011)	IndiaCommunityRural (slum)	Cross-sectional	15–49 yrsEver married in-migrantsN = 965		Use sanitary pads during menstruation	Reused cloth during menstruation		Self-reported vaginal discharge		OR: 25.07^3^(9.6–65.2)p<0.001			None	
Bhatia (1995)	India,Community Rural	Cross-sectional	<35 yrsWith one child<5yoN = 3600		‘Good’ hygiene index score^2^	‘Poor’ hygiene index score^2^		Self-reported discharge+ bad odour, itching/irritation		OR: 1.49(No CI)^4^P<0.05			Socio-economic, demographic, obstetric history, contraceptive use	
Wasserheit (1989)	BangladeshCommunityRural	Cross-sectional	15–44 yrsNon-pregnant, using contraceptionN = 2929		Stay at home and use corner of saris	Use rags washed in river and dried at home		Clinically confirmed BV (Amsel criteria); VVC or Trichomoniasis		OR: 1.74(1.33–2.27)P<0.05			Current birth control method, prior birth control, duration of current birth use	
Sami (2012)	PakistanHospitalUrban	Case-control	24–34 yrs> = 1 conception and tryingN = 800		Sanitary pads/new cloth/washed cloth dried in sunlight	Cotton/unwashed rags/rags washed but dried indoors		Secondary infertility^7^		OR: 9.0(5.0–16.4)P<0.05			Age	
***Negative Association***														
Bahram (2009)	IranHealth centreUrban	Cross-sectional	15–45 yrsMarried, womenN = 500		Using sanitary pad always or sometimes	Never using sanitary pads		Clinically confirmed BV (Nugent score)		OR: 0.20^3^(0.13–0.33)P<0.001		None		
***No Association***														
Xia (2004)	ChinaCommunityRural	Cross-sectional	18–49 yrsMarried or ever-marriedN = 606	Only ever used sanitary towel		Has ever used other materials		At least one self-reported RTI symptom (discharge, itching, pain) in last 6 months	OR: 1.24(0.53–2.87)p>0.05		Age, education, ethnicity, job, husbands job, economic status, sexual attitudes and practices, RTI knowledge			
Xia (2004)	ChinaCommunityRural	Cross-sectional	18–49 yrsMarried or ever-marriedN = 606	Undergarment sun-dried every time		Undergarment sun-dried sometimes or never			OR: 0.89(0.42–1.89)p>0.05					
Narayan (2001)	IndiaSchoolUrban + Rural	Cross-sectional	12–17 yrsSchoolgirlsN = 619	‘High’ hygiene index score^2^		‘Medium’ hygiene index score		Self-reported white discharge	OR: 1.8(0.8–4.0)p>0.05		Socio-economic, rural-urban residence and age			
Demba (2005)	GambiaHealth centreUrban	Cross-sectional	>18 yrsSelf reported discharge/itchingN = 227	Use of sanitary pads		Use of old cloths washed and reused		Clinically confirmed BV (Nugent score)	OR: 1.36^3^(0.78–2.38)P = 0.2766		None			
Singh (2011)	IndiaCommunityRural (slum)	Cross-sectional	15–49 yrsEver married, in-migrantsN = 965	Use of sanitary pads		Use of fresh cloth		Self-reported vaginal discharge	OR: 1.08^3^(0.7–1.7)P = 0.7338		None			
Singh (2011)	IndiaCommunityRural (slum)	Cross-sectional	15–49 yrsEver married in-migrantsN = 965	Use of sanitary pads		Use of home-made pads		Self-reported vaginal discharge	OR: 0.70(0.37–1.32)P = 0.2688		None			
Bulut (1997)	TurkeyHealth centreUrban	Cross-sectional	15–44 yrsEver used contraceptionN = 918	Use of disposable protection		Use of old cloths washed and reused		Self reported menstrual disorders	OR: 1.42(0.89–2.25)P = 0.14		None			
Bulut (1997)	TurkeyHealth centreUrban	Cross-sectional	15–44 yrsEver used contraceptionN = 918	Use of disposable protection		Use of old cloths washed and reused		Self reported RTI	OR: 1.33(0.91–1.95)P = 0.1363		None			
Bulut (1997)	TurkeyHealth centreUrban	Cross-sectional	15–44 yrsEver used contraceptionN = 918	Use of disposable protection		Use of old cloths washed and reused		Self reported pelvic relaxation	OR: 0.97(0.59–1.59)P = 0.9096		None			
Bulut (1997)	TurkeyHealth centreUrban	Cross-sectional	15–44 yrsEver used contraceptionN = 918	Use of disposable protection		Use of old cloths washed and reused		Self reported urinary tract infection	OR: 1.17(0.74–1.85)P = 0.4999		None			
Bulut (1997)	TurkeyHealth centreUrban	Cross-sectional	15–44 yrsEver used contraceptionN = 918	Use of disposable protection		Use of old cloths washed and reused		Self reported anaemia	OR: 1.09(0.72–1.64)P = 0.6963		None			
Ali (2007)	PakistanHospitalUrban	Case-control	20–37 yrsAllN = 800	Sanitary pads/new cloth/washed cloth sun dried		Cotton/unwashed rags/rags washed but dried indoors		Secondary infertility^5^	Unspecified but non-significant		Unspecified			
Morison (2001)	GambiaHealth centreUrban	RCT - Crossover	20–53 yrsMarried, no contraceptionN = 30	Sanitary Pads		Old cloth, washed and reused		Clinically confirmed BV (Nugent score)	OR:0.69^6^(0.47–1.03)		None (RCT design)			

1 Best estimate provided i.e. adjusted if available, otherwise unadjusted, see tables 1, 2, 3 for quality assessment; ^2^Authors created ‘Hygiene Index’ from composite score on scales for knowledge, attitudes and practices; ^3^Measure of effect not provided in paper, calculated separately by reviewers; ^4^Confidence interval not reported in paper and not possible to calculate from provided data; ^5^Unable to achieve clinical pregnancy after 12 months of unprotected sex; ^6^Direction of quote measure of effect converse of that quoted in paper.

The majority of the papers looking at RTI (both self-reported and confirmed) reported one or more statistically significant association with RTI and ‘worse’ MHM, as specifically defined in each study and in the specific populations in each study (7/11). Final ORs presented (or calculated by review authors) were in the range 1.34 to 25.07. Three studies contradicted these findings and found no association. [7], [29], [30] One study found the reverse, a statistically significant association between the use of pads and RTI i.e. a negative effect of pad use or ‘better’ MHM. [35] The single intervention study reported a non-significant increase in BV (i.e. indicating a negative association) following cross-over of MHM methods from ‘traditional’ to ‘modern’ OR: 1.44 (95% CI: 0.97–2.12, p = 0.066).

**Figure 3:**
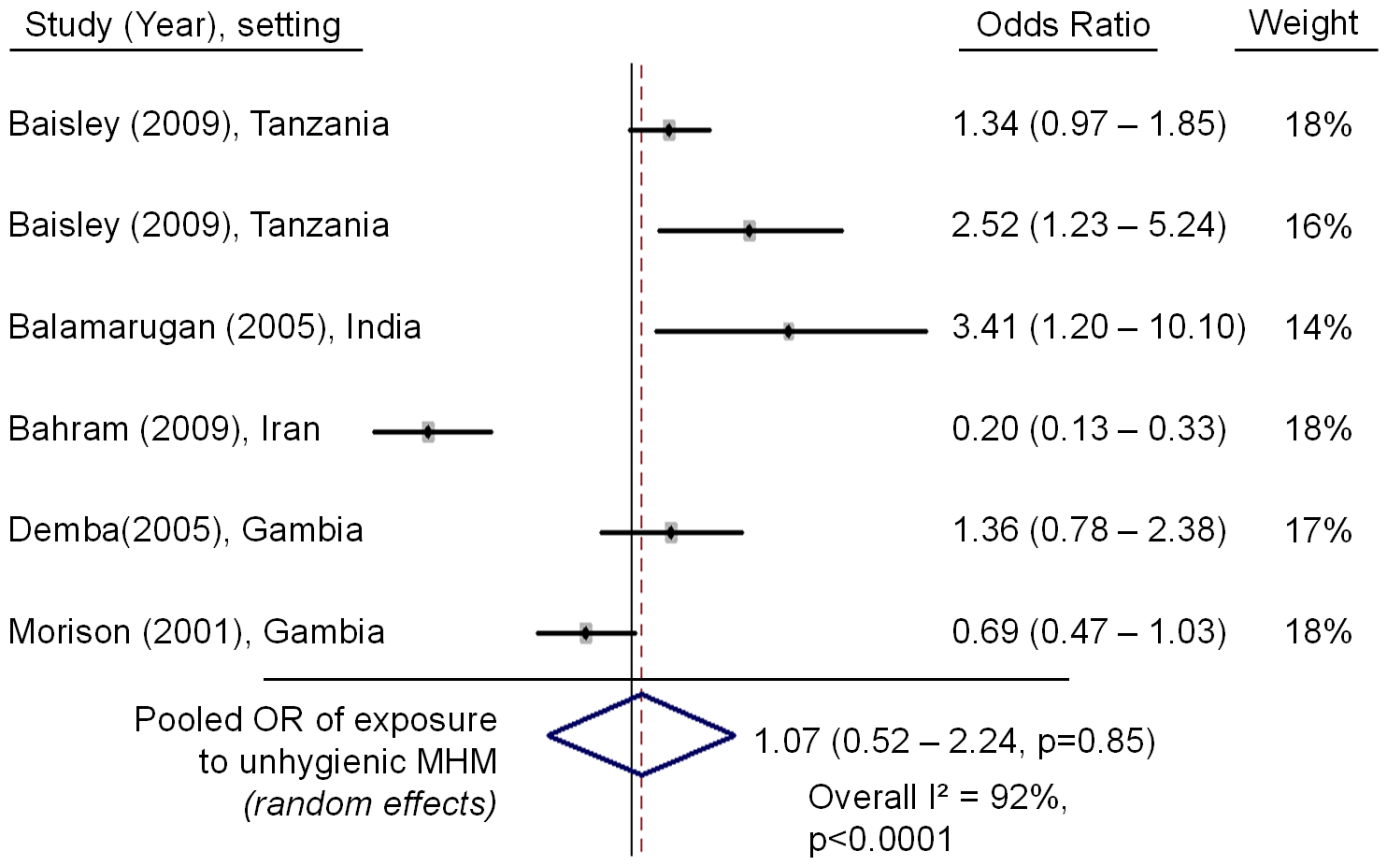
Forest plot of odds ratios of using ‘poor’ menstrual absorbent vs. ‘good’ menstrual absorbents in those with confirmed bacterial vaginosis.

By taking only higher quality studies, those which used clinically confirmed BV as an outcome measure and menstrual absorbents as an exposure, we found that of five studies, only two found an increased prevalence of BV and some specific element of ‘poor’ MHM (2/5). [4], [32] One reported an inverse relationship [35] and two, including the only randomised study, found no association. [7], [29] The odds ratios are presented in a forest plot (figure 3). The final pooled OR is 1.07 (0.52–2.24, p = 0.85) demonstrating, by this calculation, no effect. The wide variation in results and high I^2^ statistic (92%) show the high heterogeneity in this sub-group of study results.

The body of evidence to support the link between poor MHM and other health outcomes (secondary infertility, urinary tract infections and anaemia) is weak and contradictory. Two studies investigated the association between the unclean absorption of menstrual blood and secondary infertility [33], [34]. It was found that whilst the use of unclean materials for absorption of menstrual blood was not associated in one of the studies [33], the use of inappropriate material for absorption was associated with a nine-fold increase in the odds of secondary infertility in the other. [34] Of the studies investigating urinary tract infection and anaemia no studies reported an association [30], [36], [37] although prevalence of anaemia was found to be higher for those with poor MHM on one study. [30]

### Effects on Psycho-Social Outcomes including Education

#### Study design, setting and population

Of the 11 identified studies which reported psycho-social outcomes two were primarily qualitative using participatory approaches, focus groups and interviews. [18], [38] Whilst a number of other qualitative studies were identified in the search these were excluded on the basis that they did not report findings related to management, only to challenges faced as a result of menstruating. Two large cross sectional studies were identified both of which randomly selected participants. [6], [39] Six software (education) intervention studies; and one hardware (‘mooncup’) intervention study were identified. Four of these were one arm ‘before and after’ intervention studies and three had a control group of some description. Only one of these randomly allocated participants and none were blinded.

Studies were universally aimed at girls in the early stages of their menstruation (between 10 and 24 years) and predominantly based in schools. There was a good mix of urban and rural evidence. Studies were conducted in the Indian sub-continent (5/11), the Middle East or North African context (3/11); and Sub-Saharan Africa (3/11).

#### Outcomes and exposures

Qualitative studies aimed to elucidate the challenges faced in the management of menstruation in the low income context. The mooncup intervention study reported on the effect that menstruation management could have on attendance at school through matching menstruation diaries with school absenteeism records and school timetables and followed this up with the randomised distribution of the mooncup and tracked its effect on school attendance. [40] Two studies evaluated the impact of education programs on reductions in socially restrictive practices. [19], [41] Six papers reported on the effect of provision of education programs on MHM practices including use of appropriate absorbents. [19], [41]–[45] One cross-sectional study looked at the associations between use of absorbents and attendance at school [39] and another looked at associations between pre-menarcheal training and the use of sanitary pads during menstruation in later life.

#### Quality of studies and risk of bias

Standardised quality assessment summaries are provided in table 1 for intervention studies and table 3 for cross-sectional studies.

There are major quality issues around studies associating menstrual method with attendance at education or other social restrictions that do not adjust or account for socio-economic status or parents’ education in analysis or study design. Of the nine quantitative studies identified researching social effects of menstrual management or interventions, none adequately accounted for either factor. Despite the fact that those girls who are rural or in the lower socio-economic groups are reportedly most at risk of poor menstrual hygiene only two studies recruited outside of educational establishments.

Studies that aim to measure school attendance face the challenge of effectively measuring attendance. Attendance records in schools are often poorly kept or sometimes taken by other students who may cover for friends; girls may report ‘illness’ so it is hard to attribute menstruation as the reason for absence; girls newly menstruating may be irregular for a year or two which can make tracking absences difficult; and girls may be absent for a few hours and return, an outcome that won’t be captured by daily attendance records.

Randomisation, adequate control groups and blinding were lacking in the majority of the intervention studies. Two of the identified studies randomised students to receive the intervention but incorrectly state that this removes the need for base-lining their knowledge (i.e. only doing one, post-intervention, test). [42], [43] One of these studies illustrates this point by showing that the groups were not equal with their control group being younger, with an earlier menarche and with less well educated parents, all potentially better explanations for their knowledge of menstruation or behaviour than the intervention. [42] One study improved the standard pre-test/post-test comparison design by truly randomising the participants and by providing a power calculation, the only study to do the latter. [44]

In terms of statistical methods, the standard approach across most studies was to calculate the difference in test scores achieved by control and intervention groups, or to pre- and post- test only the intervention groups and test the statistical significance of any difference found using a test of difference. [42] These approaches may not always be a valid comparison as test scores are unlikely to be normally distributed. In addition one study matched recruitment but did not perform a matched analysis. [43] A number of studies failed to report associations between the intervention and individual outcomes which would have provided a greater insight into our research questions; instead they report composite measures of hygiene behaviours or knowledge which offer little insight into the specific effect of the intervention. [42]–[44]

The intensity of educational interventions also vary significantly ranging from a three year program of monthly meetings [45] to a single two hour session [42] making it difficult to draw cross-study conclusions from these studies.

#### Measures of Effect

The results of observational studies looking at social outcomes are summarised in table 5 and intervention studies are summarised in table 6.

**Table 5:** The impact of interventions on menstrual practices and social restrictions (observational studies).

First Author (year)	Setting	Design	Population and sample size	Exposure}(Baseline)	Exposure(Comparator)	Outcome	Measure of Effect^1^	Adjustments
***Positive association***								
Aniebue (2009)	NigeriaSchoolUrban	Cross-sectional	10–19 yrs	Pre-menarcheal training	No pre-menarcheal training	Self reported use of sanitary pad	1.91^2^(1.29–2.81)P = 0.001	None
Aniebue (2009)	NigeriaSchoolUrban	Cross Sectional	10–19 yrs	Pre-menarcheal training	No pre-menarcheal training	Self report menstruation has no effect on school/social life	2.02^2^(1.33–3.08)P = 0.0008	None
Ali (2010)	PakistanSchoolUrban	Cross Sectional	13–19 yrs	Attendance at school (govt./private)	Non-attendance at school	Self reported use of sanitary pad/clean cloth	2.34^2^(1.78–3.06)P<0.0001	None
***No association***								
Ali (2010)	PakistanSchoolUrban	Cross Sectional	13–19 yrs	Attendance at school (govt./private)	Non-attendance at school	Restrict socialisation during menstruation	0.82^2^(0.65–1.04)P = 0.1059	None

1 Best estimate provided i.e. adjusted if available, otherwise unadjusted, see tables 1, 2, 3 for quality assessment; ^2^Measure of effect not provided in paper, calculated separately by review authors.

**Table 6:** The impact of interventions on menstrual practices and social restrictions (intervention studies).

First Author (year)	Setting	Design	Population	Intervention	Control	Outcome(s)	Measure of effect	Adjustments
***Positive association***								
Fetohy (2007)	Saudi ArabiaSchoolUrban	Quasi-experimentalCluster randomised	14–17 yrsIn schoolN = 248	Education program(1×2 hours)	No education program	Menstrual knowledge	Improved^1^ in intervention (p<0.001)^2^	None
						Attitude toward menstruation	Improved in intervention (p<0.001)	
						Self-reported hygiene behaviour	Improved in intervention (p<0.001)	
Nemade (2009)	IndiaSchoolUrban	Non- randomised before and after	10–19 yrsIn schoolN = 217	Education program(1×‘lecture’)	No education program	Self reported menstrual hygiene practices	Significant improvement in good pad washing (+38%); drying (+28%)	None
						Self reported social restrictions	No reported effect	
Allah (2011)	EgyptSchoolUrban	Multistage random sampling	14–16 yrsIn schoolN = 150	Education program(4×45 minutes)	No education program	Menstrual knowledge	Improved in intervention (p<0.001)^3^	None
						Restricted household activities	Non-significant reduction from 56% to 44% (p = 0.12)	
						Frequency of using, changing, washing pads with soap and drying in sun	Improved in intervention (p<0.001)	
						Disposal of used pads	Improved in intervention (p<0.001)	
Posner (2009)	NepalCommunityRural	Non- randomised before and after	11–24 yrsAll girlsN = 504	Peer education training(2×8 hours + 24 weekly sessions)	No peer educator training	Social restrictions practiced including food and religious	Mean reduction of one restriction (p<0.001). Proportion reduction of 18% for religious and 6% for food (p<0.001). 23% ceased all restrictions	Stratified results
Dongre (2007)	IndiaCommunity Rural	Non- randomised before and after	12–19 yrsAll girlsN = 383 girls	Education program(36× meetings)	No education program	Awareness of menstruation	Increase 35% to 55% (p<0.05)	None
						Pad use	Increase in 5% to 25% (p<0.05)	
						Cloth reuse	Decrease 85% to 57% (p<0.05)	
						Dietary Restrictions	Decrease 56% to 48% (p>0.05)	
***No association***								
Fakhri (2012)	IranSchoolRural/urban	Quasi-experimentalNon -random	14–18 yrsIn educationN = 698	Education program(10×2 hours)	No education program	Pad material; changing frequency; social restrictions including diet; school attendance; bathing habits	8.6% rated as having ‘good/excellent’ hygiene in intervention vs. 4.9% in control. No test of difference or individual reported measures	None
Oster (2010)	NepalSchoolUrban	Randomised controlled trial	12–16 yrsN = 198	Menstrual barrier ‘moon cup’	No mooncup	School absenteeism	No significant difference to school attendance following intervention	None

1 As only composite scores are given we cannot determine in which specific elements improvements were made between groups; ^2^T-Test; ^3^McNemar Test.

Two observational studies were found which presented sufficient analysis to associate menstrual education (software), either received at school or at home, with improved menstrual practices. [6], [39] One studied the impact of pre-menarcheal training and found that around half of the menstruating girls in a Nigerian school had received some form of pre-menarcheal training, primarily from their mothers. Those who had received this training were more likely to use pads than toilet roll or cloth; more likely to dispose of their used absorbents hygienically; and more likely to report that menstruation had ‘no effect’ on their social lives. This was supported by research from Pakistan which found that menstruating girls attending formal education were less likely to be using unhygienic absorbents. [39] This finding however may also be viewed as evidence for the role effective management might have on school attendance rather than vice versa. These papers are supported by two qualitative studies which reported findings on the impact management has on attendance at school. One third of the schoolgirls in a study in Tanzania reported lack of pads as a reason for absenteeism. 43% felt they did not have enough privacy to manage their menstruation at school, and that it was not possible to wash and dry pads in the open due to cultural limitations. [18] A third of respondents in another qualitative study reported missing school due to the lack of suitable absorbents. [38]

All of the education intervention studies bar one reported a positive change in MHM behaviours following a program of education. Improvements varied from study to study but included a significant difference in bathing during menstrual period between those receiving the intervention vs. the control group; [43] a five-fold increase in pad use; [45] and a 21% increase in the proportion of girls hygienically washing their menstrual pads. [41] One study reported improved knowledge amongst those who had attended a program but did not track their behaviour. [42] One study did not report its findings in a way that demonstrated a significant improvement in behaviour although there was a stated improvement. [43] Overall the quality of the evidence indicating that providing targeted education can improve MHM practices such as the use of disposable absorbents, changing and washing of pads was consistent and persuasive.

Six educational intervention studies investigated the impact education can have on the practicing of social restrictions including limiting diet; avoiding specific household chores; and missing school. All of the studies reported that such restrictions were present in their study sites. Only one study found a significant improvement in restrictions practiced with a mean reduction of one restriction per participant following a long and involved peer education training program. The study found a more marked effect where restrictions were individual in nature and did not involve ‘polluting’ others e.g. more girls abandoned ‘sleeping outside’ than abandoned ‘not cooking for others’ because they felt it would only affect themselves. The study also found that restrictions practiced varied greatly by religion, caste and education with religious restrictions the hardest to overcome. About a quarter of participants in the study reported abandoning all restrictions. [19] Two additional studies reported smaller (non-significant) reductions in restrictive practices such as limiting diet during menstruation [45] and avoiding ‘household activities’ during menstruation. [44] Two studies contradicted these findings and reported no effect of education on restrictions practiced. [41], [43] Overall the strength of evidence that education programs can change the practice of social restrictions was moderate and strongly dependent on: the context; the type of restriction practiced; and the quality (including length) of the intervention.

The only identified trial of a hardware intervention (the ‘mooncup’) reported no effect on school attendance and furthermore reported that there was very little scope for reducing absenteeism in those who were already attending school. They calculated the difference between absenteeism during a girl's menstrual period vs. non-menstrual period to be less than one day per year and the difference between attendance of girls who were randomly allocated to receive a mooncup and those who did not as insignificant. [40] No further evidence was found regarding the effect of menstrual management on attendance at school or rates of drop-out. No evidence was found regarding the impact menstrual hardware can have on other social restrictions practiced.

## Discussion

Our review sought to identify literature investigating the effects of MHM on health and psycho-social outcomes. Our search was open to a number of potential biases and it is important to be clear about the effect these may have had on the results we have reported.

It was our intention that by setting no time limit on our search, using broad search terms, and including three major on-line repositories we would minimise the potential for any literature selection bias. An additional step was taken in hand searching identified articles for relevant references. This returned six additional studies, three of which were not available online but only in hard copy journals. Overall we feel that we spread our search as wide as was feasible given our time and resource constraints. A major challenge in our search lay in the decision to not include unpublished research. In a relatively poorly researched field such as MHM there is a strong possibility that the best knowledge lies in the hands of those implementing programs; working at non-governmental organisations or in informal research. We acknowledge the potential for this bias and would urge any future reviewers to endeavour to widen the search to include these forms of literature where resources allow.

We have sought to minimise any reviewer bias by undertaking the review in partnership with the relevance, findings and quality of each paper assessed by two reviewers and the results compared and discussed. Selective reporting bias or publication bias is a possibility in any review. It is unfortunately the case that positive research is more often published than that with null or negative findings. In our review we identified a wide spread of results ranging from strongly positive relationships to the converse. Many of our included studies were often only secondarily concerned with menstrual hygiene and we believe this demonstrates that reluctance to publish null results was of only minimal concern to researchers in this topic. Finally, three potentially relevant studies were rejected due to being in a language other than English. Given the resource we would have sought to widen our review to include these papers but this again was not possible given the time and resource available.

With these limitations in mind, it is our conclusion that the weight of the research that was identified in relation to menstrual management lies in the background, in the establishment of the prevalence of the exposure. There are numerous papers looking at menstrual knowledge, awareness and practice in specific low income contexts. Although each study deals in detail with a specific setting where factors vary, one thing is clear: menstruation is poorly understood and poorly researched. The papers identified and reviewed do not currently allow us to understand the ways in which existing methods of MHM impact on women and girl's health or freedoms or the extent to which improving menstrual management would improve lives.

In this review the majority of studies looking at the impact of MHM on health reported that poor MHM, mainly the use of less hygienic absorbents, was related with RTI. The methodological shortcomings of the health research were many however including: lack of adjustment for confounding, in particular socio-economic status and sexual activity; limited discussion of the problem of concurrent infection; lack of specificity in case definitions; and reliance on observational evidence. These limitations mean that we cannot draw strong conclusions regarding our first research question. The highest quality evidence, that which employed some objective measure of bacterial vaginosis, reported a very mixed set of results and resulted in a pooled measure of no effect. The high heterogeneity found in the study results most likely reflects the wide variation in approaches used by researchers even when facing the same research question and using broadly similar case definitions. We can therefore report that there is an initial indication that MHM may be associated with an increased risk of RTI but the strength and route of infection is not known. More research, and specifically more methodologically consistent research, is required in the area of RTI and MHM.

In terms of the associations between menstrual management and women's and girls' social and psychological well-being and development, from this review it appears likely that education programs have some effect on preparation for menstruation and can improve menstrual practices in at least some groups of girls: most likely those already in education. The failure of researchers to randomise participants; include girls both in and out of education; or adjust in the analysis for parents' education or socio-economic status; mean it is difficult to know whether this effect would hold across other settings and groups. We can however report that there is evidence for the effectiveness of educational programs in improving menstrual knowledge and management.

The body of evidence provides us with little or no evidence regarding the management characteristics of those who practice restrictions vs. those who do not practice restrictions. This leaves us asking ‘are those who have better MHM less likely to practice restrictions, and why?’ For example, it is plausible that the use of pads would reduce the chance of spotting or smell but not feasible that it would reduce pain experienced which may also contribute to school absenteeism during days of menstruation.

Despite the apparent acceptance in WASH policies that menstrual management affects attendance of adolescent girls at school there is very little high quality evidence associating school attendance or drop-out with menstrual management. The only published study identified found no association between provision of a menstrual cup and school attendance. [40] An unpublished study by Scott et al found significant improvements of 9% to 14% in recorded class attendance from access to sanitary napkins and/or MHM education but full details of the study methods and results were not available at the time of the review. [46] A systematic review into the linkages between separate toilets for girls and school attendance was inconclusive. The data were analysed without taking account of age with respect to menstruation and MHM provisions in school may have been among the influencing factors. [47] No studies were found which addressed provision of pain medication or other factors that may have a bearing on attendance or drop-out rates. We cannot therefore report that the current evidence indicates improved MHM improves attendance at school. More research is needed on the reasons for school absenteeism of adolescent girls including those beyond MHM and if and how provision of absorbents or other interventions can be cost-effective and sustainable. Where studies have been conducted, for example the trial of the mooncup in Nepal, it is likely that the context has had a strong effect on the outcome. As no absenteeism problem was identified at the outset of that study the results of the intervention trial itself are somewhat muted. [39]

Our review excluded those studies looking at tampon use but a valid question remains: how would tampon use impact on outcomes studied in lower income countries? There is a comprehensive body of evidence investigating potential associations between the use of mass manufactured sanitary products and health outcomes including toxic shock syndrome (TSS) and dermatological complaints in high income countries. TSS is an extremely rare outcome and of little relevance to the majority of women in the countries of interest for this report as far as we know. A comprehensive review of the evidence found that external absorbent ‘liners’ are safe when used as intended and do not promote VVC or urinary tract infections. [48] This review was supported in its conclusions on VVC and BV by a recent high quality RCT. [49] As tampons are increasingly promoted in low-income countries it will be important to remain vigilant as to the possible health consequences of use in conditions of poor hygiene and potentially less frequent changing.

One population of particular interest for further study would be to investigate the network of effect for people living with HIV/AIDS in light of the potential associations between menstrual protection, reproductive tract infections and HIV status. Recent systematic reviews have highlighted the potential for prompt treatment of BV and VVC as a route to reduce rates of HIV infection, could prevention of BV through improved menstrual management also have a role to play? [13], [16]

### Implications for Future Practice and Research

In conclusion, there is much still to be done to build the evidence base. Raising awareness regarding menstruation and hygienic practices has remained largely a neglected area in terms of research, despite its increasing popularity amongst public health organisations.

With this review we hope we have provided some basis for those planning future research in this area. Our aim was to collate the available evidence and to critically appraise it not for purely academic purposes but to highlight the strengths and weaknesses of studies related to this topic and to motivate other researchers to improve future efforts.

We believe that there is much scope for dedicated menstrual hygiene research and that of primary importance is an agreed theory of the effects of poor menstrual hygiene management amongst researchers in the field. Once this is in place a multidisciplinary effort should be made to better understand this wide-reaching issues which is of relevance to many millions of women and girls across the world.

## References

[1] Jones LL, Griffiths PL, Norris SA, Pettifor JM, Cameron N (2009) Age at menarche and the evidence for a positive secular trend in urban South Africa. Am J Hum Biol 21: 130–132. 18942702 10.1002/ajhb.20836

[2] Sharma K (1990) Age at menarche in northwest Indian females and a review of Indian data. Ann Hum Biol 17: 159–162. 2334111 10.1080/03014469000000912

[3] Walker AR, Walker BF, Ncongwane J, Tshabalala EN (1984) Age of menopause in black women in South Africa. Br J Obstet Gynaecol 91: 797–801. 6466582 10.1111/j.1471-0528.1984.tb04853.x

[4] Baisley K, Changalucha J, Weiss HA, Mugeye K, Everett D, et al (2009) Bacterial vaginosis in female facility workers in north-western Tanzania: prevalence and risk factors. Sexually Transmitted Infections 85: 370–375. 19473997 10.1136/sti.2008.035543 PMC2709714

[5] Adinma ED, Adinma JI (2008) Perceptions and practices on menstruation amongst Nigerian secondary school girls. African Journal of Reproductive Health 12: 74–83. 20695158

[6] Aniebue UU, Aniebue PN, Nwankwo TO (2009) The impact of pre-menarcheal training on menstrual practices and hygiene of Nigerian school girls. Pan Afr Med J 2: 9. 21532905 PMC2984277

[7] Demba E, Morison L, Loeff MSvd, Awasana AA, Gooding E, et al (2005) Bacterial vaginosis, vaginal flora patterns and vaginal hygiene practices in patients presenting with vaginal discharge syndrome in The Gambia, West Africa. BMC Infectious Diseases 5. 10.1186/1471-2334-5-12 PMC1083415 15757510

[8] Dasgupta A, Sarkar M (2008) Menstrual hygiene: how hygienic is the adolescent girl? Indian Journal of Community Medicine 33: 77–80. 19967028 10.4103/0970-0218.40872 PMC2784630

[9] Narayan K, Srinivasa D, Pelto P, S V (2001) Puberty Rituals, Reproductive Knowledge and Health of Adolescent Schoolgirls in South India. Asia-Pacific Population Journal 16: 225–238.

[10] Wasserheit JN, Harris JR, Chakraborty J, Kay BA, Mason KJ (1989) Reproductive tract infections in a family planning population in rural Bangladesh. Stud Fam Plann 20: 69–80. 2785722

[11] Bhatti LI, Fikree FF (2002) Health-seeking behavior of Karachi women with reproductive tract infections. Social Science & Medicine 54: 105–117. 11820674 10.1016/s0277-9536(01)00012-0

[12] Sweet RL (2000) Gynecologic conditions and bacterial vaginosis: implications for the non-pregnant patient. Infect Dis Obstet Gynecol 8: 184–190. 10968604 10.1155/S1064744900000260 PMC1784684

[13] Atashili J, Poole C, Ndumbe PM, Adimora AA, Smith JS (2008) Bacterial vaginosis and HIV acquisition: a meta-analysis of published studies. AIDS 22: 1493–1501. 18614873 10.1097/QAD.0b013e3283021a37 PMC2788489

[14] Gillet E, Meys JF, Verstraelen H, Bosire C, De Sutter P, et al (2011) Bacterial vaginosis is associated with uterine cervical human papillomavirus infection: a meta-analysis. BMC Infect Dis 11: 10. 21223574 10.1186/1471-2334-11-10 PMC3023697

[15] Ugwumadu AH (2002) Bacterial vaginosis in pregnancy. Curr Opin Obstet Gynecol 14: 115–118. 11914687 10.1097/00001703-200204000-00003

[16] Johnson LF, Lewis DA (2008) The effect of genital tract infections on HIV-1 shedding in the genital tract: a systematic review and meta-analysis. Sex Transm Dis 35: 946–959. 18685546 10.1097/OLQ.0b013e3181812d15

[17] McMahon SA, Winch PJ, Caruso BA, Obure AF, Ogutu EA, et al (2011) 'The girl with her period is the one to hang her head' Reflections on menstrual management among schoolgirls in rural Kenya. BMC Int Health Hum Rights 11: 7. 21679414 10.1186/1472-698X-11-7 PMC3129305

[18] Sommer M (2010) Where the education system and women's bodies collide: The social and health impact of girls' experiences of menstruation and schooling in Tanzania. Journal of Adolescence 33: 521–529. 19395018 10.1016/j.adolescence.2009.03.008

[19] Posner J, Kayastha P, Davis D, Limoges J, O'Donnell C, et al (2009) Development of leadership self-efficacy and collective efficacy: adolescent girls across castes as peer educators in Nepal. Glob Public Health 4: 284–302. 19437216 10.1080/17441690902783157

[20] Piper-Pillitteri S (2011) School menstrual hygiene management in Malawi: More than toilets. London, UK: WaterAid.

[21] Anandalakshmy S (1994) The Girl Child and the Family: An Action Research Study. New Delhi, India: Indian University and Ministry of Human Resources Development.

[22] Bharadwaj S, Patkar A (2004 ) Menstrual hygiene and management in developing countries: Taking stock. , Mumbai, India: Junction Social.

[23] Kanyemba A ( 2011) Growing up at school: A guide to menstrual management for school girls. Pretoria, South Africa: Water Research Commission.

[24] Carrard N, O'Riordan K (2011) Menstrual Hygiene Management. Canberra, Australia: Civil Society WASH learning Fund.

[25] Bility K, Onya H (2000) Water use, sanitation practices, perceptions and hygiene education in primary school children in the Northern Province and Western Cape. Pretoria, South Africa: Water Research Commission.

[26] UNICEF (2004) The State of the World's Children 2004 - Girls, education and development.

[27] Harris RJ, Bradburn MJ, Deeks JJ, Harbord RM, Altman DG, et al (2008) metan: fixed- and random-effects meta-analysis. The Stata Journal 8: 15.

[28] Moher D, Liberati A, Tetzlaff J, Altman DG (2010) Preferred reporting items for systematic reviews and meta-analyses: the PRISMA statement. Int J Surg 8: 336–341. 20171303 10.1016/j.ijsu.2010.02.007

[29] Morison L, Ekpo G, West B, Demba E, Mayaud P, et al (2005) Bacterial vaginosis in relation to menstrual cycle, menstrual protection method, and sexual intercourse in rural Gambian women. Sexually Transmitted Infections 81: 242–247. 15923295 10.1136/sti.2004.011684 PMC1744975

[30] Bulut A, Filippi V, Marshall T, Nalbant H, Yolsal N, et al (1997) Contraceptive choice and reproductive morbidity in Istanbul. Stud Fam Plann 28: 35–43. 9097384

[31] Singh MM, Devi R, Garg S, Mehra M (2001) Effectiveness of syndromic approach in management of reproductive tract infections in women. Indian Journal of Medical Sciences 55: 209–214. 11665391

[32] Balamurugan SS, Bendigeri ND (2012) Community-based study of reproductive tract infections among women of the reproductive age group in the urban health training centre area in Hubli, Karnataka. Indian Journal of Community Medicine 37: 34–38. 22529538 10.4103/0970-0218.94020 PMC3326805

[33] Ali TS, Sami N, Khuwaja AK (2007) Are unhygienic practices during the menstrual, partum and postpartum periods risk factors for secondary infertility? J Health Popul Nutr 25: 189–194. 17985820 PMC2754005

[34] Neelofar S, Ali TS, Saba W, Saleem S (2012) Risk factors for secondary infertility among women in Karachi, Pakistan. PLoS ONE 7. 10.1371/journal.pone.0035828 PMC3338792 22558233

[35] Bahram A, Hamid B, Zohre T (2009) Prevalence of bacterial vaginosis and impact of genital hygiene practices in non-pregnant women in zanjan, iran. Oman Med J 24: 288–293. 22216382 10.5001/omj.2009.58 PMC3243866

[36] Younis N, Khattab H, Zurayk H, el-Mouelhy M, Amin MF, et al (1993) A community study of gynecological and related morbidities in rural Egypt. Stud Fam Plann 24: 175–186. 8351698

[37] Bhatia JC, Cleland J (1995) Self-reported symptoms of gynecological morbidity and their treatment in south India. Stud Fam Plann 26: 203–216. 7482678

[38] Crofts T, Fisher J (2012) Menstrual hygiene in Ugandan schools: an investigation of low-cost sanitary pads. Journal of Water, Sanitation and Hygiene for Development 2: 50–58.

[39] Ali TS, Rizvi SN (2010) Menstrual knowledge and practices of female adolescents in urban Karachi, Pakistan. Journal of Adolescence 33: 531–541. 19589587 10.1016/j.adolescence.2009.05.013

[40] Oster E, Thornton R (2011) Menstruation, Sanitary Products and School Attendance: Evidence from a Randomized Evaluation. American Economic Journal: Applied Economics 3

[41] Dipali N, Seema A, Rupali G (2009) Impact of health education on knowledge and practices about menstruation among adolescent school girls of Kalamboli, Navi-Mumbai. Health and Population Perspectives and Issues 32: 167–175.

[42] Fetohy EM (2007) Impact of a health education program for secondary school Saudi girls about menstruation at Riyadh city. J Egypt Public Health Assoc 82: 105–126. 18217327

[43] Fakhri M, Hamzehgardeshi Z, Golchin NAH, Komili A (2012) Promoting menstrual health among Persian adolescent girls from low socioeconomic backgrounds: a quasi-experimental study. BMC Public Health 12.. 10.1186/1471-2458-12-193 PMC3348061 22420743

[44] Allah ESA, Elsabagh EEM (2011) Impact of health education intervention on knowledge and practice about menstruation among female secondary school students in Zagazig city. The Journal of American Science 7: 737–747.

[45] Dongre AR, Deshmukh PR, Garg BS (2007) The effect of community-based health education intervention on management of menstrual hygiene among rural Indian adolescent girls. World Health & Population 9: 48–54. 10.12927/whp.2007.19303 18272942

[46] Scott L, Dopson S, Montgomery P, Dolan C, Ryus C (2009) Impact of Providing Sanitary Pads To Poor Girls in Africa. University of Oxford.

[47] Birdthistle I, Dickson K, Freeman M, Javidi L (2011) What impact does the provision of separate toilets for girls at schools have on their primary and secondary school enrolment, attendance and completion?. A systematic review of the evidence. London: EPPI-Centre, Social Science Research Unit, Institute of Education, University of London.

[48] Farage M, Bramante M, Otaka Y, Sobel J (2007) Do panty liners promote vulvovaginal candidiasis or urinary tract infections? A review of the scientific evidence. Eur J Obstet Gynecol Reprod Biol 132: 8–19. 17204360 10.1016/j.ejogrb.2006.11.015

[49] Giraldo PC, Amaral RL, Juliato C, Eleuterio J Jr, Brolazo E, et al (2011) The effect of "breathable" panty liners on the female lower genital tract. Int J Gynaecol Obstet 115: 61–64. 21798535 10.1016/j.ijgo.2011.04.016

